# Research note: A pioneering protocol for cryopreservation of grey partridge (*Perdix perdix* L.) semen

**DOI:** 10.1016/j.psj.2026.107823

**Published:** 2026-09-21

**Authors:** Marcel Bawej, Marta Michalak, Regina Grugel, Artur Kowalczyk

**Affiliations:** Division of Poultry Breeding, Institute of Animal Husbandry and Breeding, Wrocław University of Environmental and Life Sciences, Chełmońskiego 38C, Wrocław, 51-630, Poland

**Keywords:** *Perdix perdix*, Artificial insemination, Farmbird conservation, Biobank

## Abstract

The European population of the grey partridge (*Perdix perdix* L.) in the natural environment is rapidly declining. The aim of the present study was to determine the feasibility of fertilization using cryopreserved semen. The study was conducted on one-year-old birds: ten males and four females. Semen was frozen using a modified method developed for capercaillie semen. Eight artificial inseminations were performed with cryopreserved semen, with each female inseminated twice, using a mean dose of 6.1 × 10⁶ live spermatozoa. The mean fertilization rate was 25.0%, and the duration of fertility after a single insemination was two days. The results demonstrated the possibility of using cryopreserved semen in the reproduction of the grey partridge, which may be helpful for the future conservation of this species.

## Introduction

The population of the grey partridge (*Perdix perdix* L.) in the natural environment is considered to be rapidly declining in Europe (Laux and Gottschalk, 2025). The main causes are the intensification and chemicalization of agriculture, which negatively affect populations of small farmland avifauna. The drastic decline in the partridge population has led to the adoption of various forms of protection, such as restrictions on hunting or population support through the introduction of appropriately bred individuals (Tajchman et al., 2024). *Ex situ* breeding is an important tool in conservation programs for endangered species (O’Brien et al., 2024; Witzenberger and Hochkirch, 2011). Under breeding conditions, avian reproduction usually occurs through natural mating; however, the development of reproductive technologies in breeding centers, such as artificial insemination (**AI**) and semen storage, is crucial for preserving the genetic diversity of captive populations (Prieto et al., 2018; Santiago-Moreno and Blesbois, 2022). Semen preservation is an important biotechnology used to safeguard the genetic material of birds, especially those with declining populations, through biobanking (Bezerra et al., 2024).

Considering the shrinking populations of the grey partridge in Europe, we initiated pioneering studies aimed at determining the basic characteristics of semen and the possibility of its short-term storage (Bawej et al., 2026). The aim of the present study was to determine the feasibility of fertilization using cryopreserved semen. To our knowledge, this study represents the first species-specific demonstration of functional cryosurvival and in vivo fertilizing capacity of frozen-thawed grey partridge semen.

## Materials and methods

### Experimental birds and husbandry

The study protocol was evaluated by the Local Ethical Committee for Animal Experiments in Wrocław (Resolution No. 021/2023/P1: proceedings discontinued as applied techniques fell under routine husbandry and veterinary practices) and was performed in accordance with Directive 2010/63/EU on the protection of animals used for scientific purposes.

Research was conducted during the reproductive season on fourteen one-year-old grey partridges (ten males, four females) from a single cohort. Prior to the breeding season, birds were transferred to individual outdoor enclosures (80 × 40 × 50 cm) to prevent territorial aggression. They were maintained under natural photoperiod and ambient temperature with ad libitum access to food and water, maintaining continuous visual and vocal contact.

### Semen collection and cryopreservation

Semen was collected twice a week via dorso-abdominal massage using a sterile urological catheter. Ejaculate volumes were measured, and samples were pooled and immediately diluted (1:1) in EK extender (comprising 1.4 g sodium glutamate, 0.14 g potassium citrate monohydrate, 0.7 g glucose, 0.2 g d-fructose, 0.7 g inositol, 0.1 g polyvinylpyrrolidone, 0.02 g protamine sulfate, 0.98 g anhydrous sodium hydrogen phosphate, and 0.21 g anhydrous sodium dihydrogen phosphate per 100 mL of double-distilled water; pH 7.3, osmolality 390 mOsm/kg) for initial quality evaluation. Semen cryopreservation was performed according to the straw method described by Kowalczyk and Łukaszewicz (2015) using 6% dimethylformamide (**DMF**; SERVA, Heidelberg, Germany) as a cryoprotectant. A minor modification was applied during freezing in liquid nitrogen vapor: straws were suspended 1.5 cm above the liquid nitrogen surface, which allowed for a cooling rate of approximately 60 °C/min, previously established in our laboratory using a thermocouple thermometer (Delta OHM, HD2108.1, Caselle di Selvazzano, Italy). Thawing was performed by immersing the straws in a 60 °C water bath for 3 seconds.

### Semen evaluation

Fresh and post-thaw sperm motility, progressive motility, curvilinear velocity, straight-line velocity, average path velocity, amplitude of lateral head displacement and linearity were analyzed using the Sperm Class Analyzer® (Microptic S.L., Barcelona, Spain). Samples were diluted (1:20) in EK extender, loaded onto a warmed (38 °C) 20-µm Leja 8-chamber slide (Leja Products B.V., Nieuw-Vennep, The Netherlands), and evaluated at 50 Hz (50 frames/field, 2–20 µm² particle area, connectivity 12 px and average path velocity above 5 µm/s for categorizing motile spermatozoa). Sperm viability and morphology were assessed via nigrosin-eosin smears evaluated on a heating plate (37 °C), classifying 200 cells per sample into seven morphological categories.

### AI and fertility

Following cloacal eversion, intravaginal AI was performed within 3 minutes of semen thawing using a 70-mm turkey straw rounded at both ends (IMV Technologies, L'Aigle, France) connected to an aspirator tube equipped with a mouthpiece. Four females received a total of eight inseminations (AI volume adjusted to yield 3.2 × 10⁶ to 9.1 × 10⁶ live spermatozoa per dose). Re-insemination occurred only after females began laying unfertilized eggs to ensure sperm depletion from the previous AI. Eggs were collected daily, stored at 15 °C (up to five days), and incubated at 37.8 °C (55% humidity). Fertility was assessed per AI event among eggs laid following a given AI, based on the proportion of eggs showing embryonic development, via egg breakout on the 4th day of incubation.

### Statistical analysis

Statistical analyses and data visualization were performed using R software (ver. 4.5.3, R Core Team, Vienna, Austria). A paired Wilcoxon signed-rank test was used to compare fresh and post-thaw parameters; the experimental unit was defined as the pooled ejaculate (n = 25). Differences were considered statistically significant at P < 0.05.

## Results and discussion

The mean ejaculate volume was 5.3 µL, and the average sperm concentration was 1.15 × 10⁹/mL. The cryopreservation procedure applied to grey partridge semen caused a significant decrease (P < 0.05) in sperm motility and in the analyzed movement parameters (Fig. 1). Morphological evaluation of thawed semen also revealed significant changes (P < 0.05) in the proportions of all distinguished sperm forms (Fig. 1). After eight insemination procedures performed with a mean dose of 6.1 × 10⁶ live spermatozoa, 25.0% fertilized eggs were obtained. The mean duration of fertility was two days. Detailed reproductive performance of individual females is presented in Table 1. It is important to note that due to the limited sample size and the restricted number of inseminated females, the observed 25.0% fertility rate should be interpreted as a proof-of-concept rather than an expected species-level performance. Consequently, further research involving larger cohorts and parallel fresh-semen controls is required to estimate population-level fertility rate. Moreover, while a cooling rate of approximately 60 °C/min was sufficient to obtain functional spermatozoa in this study, future protocols should investigate alternative cooling rates to better reduce osmotic shock and intracellular ice formation, as demonstrated in other Galliformes (Blesbois et al., 2008; Bezerra et al., 2024).

**Fig. 1:**
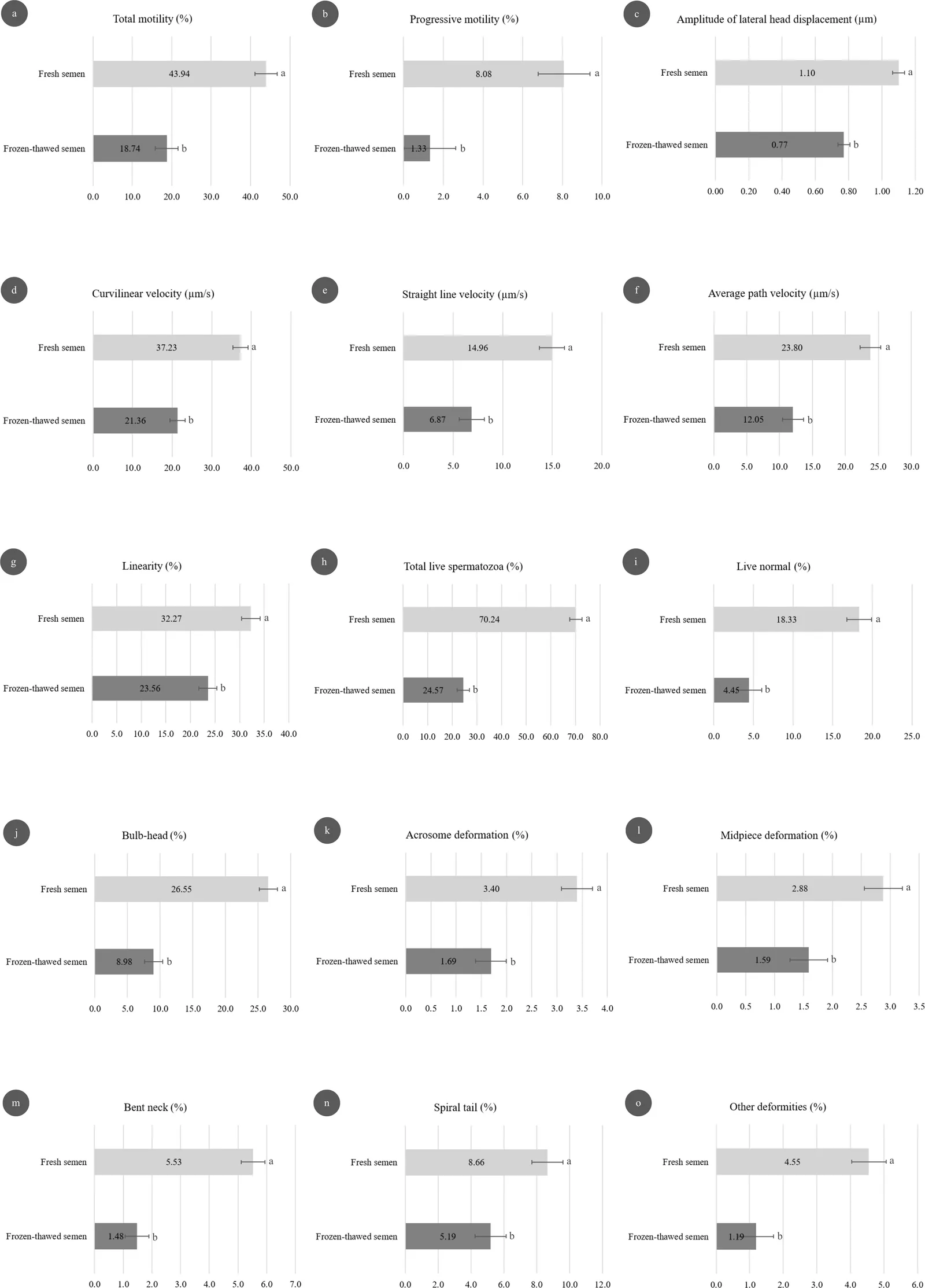
Effect of cryopreservation on the *in vitro* quality of grey partridge (*Perdix perdix* L.) spermatozoa. Bar charts compare fresh and post-thaw samples regarding motility and kinematic parameters (a–g) as well as viability and morphological characteristics (h–o). Bars represent mean ± SEM. Different letters (a, b) indicate significant differences (*P* < 0.05, paired Wilcoxon signed-rank test).

*Note: The apparent reduction in the percentages of specific morphological abnormalities in frozen-thawed semen (panels j–o) is a direct consequence of the decrease in the overall population of live spermatozoa (panel h) following cryopreservation, rather than an improvement in morphological quality*.

**Table 1:** Individual reproductive performance of grey partridge (*Perdix perdix*) following artificial insemination with frozen-thawed semen.

Female ID	No. AI	Mean sperm dose of live spermatozoa (× 10⁶)	No. eggs after AI (n)	Fertilized eggs (n)	Fertility (%)	Days with fertile eggs
F1	2	8.14	4	1	25.0	2
F2	2	4.60	4	1	25.0	2
F3	2	7.49	5	1	20.0	2
F4	2	4.32	3	1	33.3	1
Total/Average	8	6.14	16	4	25.0	1.8

Several challenges are encountered in developing effective protocols for chilling and cryopreserving avian semen, ranging from sample collection, due to high levels of contamination and very small volumes, to the selection of appropriate thawing methods (Bezerra et al., 2024). Another challenge was the unknown effect of cryoprotectants on grey partridge semen; for instance, glycerol exerts a contraceptive effect in the avian female reproductive tract (Santiago-Moreno and Blesbois, 2022), and our preliminary trials using the pellet method with dimethylacetamide (**DMA**) showed that grey partridge spermatozoa did not survive freezing. Consequently, we applied a straw cryopreservation technique using 6% **DMF**, previously described for capercaillie (*Tetrao urogallus* L.) semen, a species belonging to the same order, Galliformes (Kowalczyk and Łukaszewicz, 2015). Although this method resulted in 84.4% fertilization in capercaillie, this value cannot be treated as a direct performance threshold for the grey partridge. The small ejaculate volume, low sperm concentration, low motility, and high proportion of morphological abnormalities observed in this study reflect an inherently lower baseline semen quality compared to capercaillie, which aligns with the species-specific semen characteristics we recently established for this species (Bawej et al., 2026). In cryopreserved grey partridge semen, the relative decreases in sperm motility and in the proportion of morphologically normal spermatozoa were 57.4% and 75.6%, respectively. These changes were greater than those reported for capercaillie semen, in which the relative decreases in motility and normal sperm morphology were 43.7% and 61.8%, respectively (Kowalczyk and Łukaszewicz, 2015). The relationships observed in grey partridge semen are similar to those described by Blesbois et al. (2008) in rooster (*Gallus gallus domesticus* L.) semen. These authors demonstrated that the physical and osmotic stressors of the cryopreservation process disproportionately amplify preexisting cellular damage in semen of initially poor quality. Such baseline vulnerability makes the structural and oxidative changes induced by freezing particularly large and unfavorable, which likely explains the comparatively lower post-thaw fertilization rate in the grey partridge relative to the capercaillie.

Despite the low percentage of spermatozoa surviving the cryopreservation process, AI with an average dose of 6.1 million live spermatozoa made it possible to obtain egg fertilization at a rate of 25.0%. In all cases, fertilization was observed only in the first egg laid after AI. The persistence of fertility for two days may have resulted from the limited capacity for sperm storage in the oviduct of females. In our previous studies, we observed a short duration of fertility after insemination with fresh semen, lasting a maximum of four days, and attributed this to the monogamy of the species and its high copulation frequency, averaging 1.23 times per day (Bawej et al., 2026).

In conclusion, the proposed method for cryopreservation of grey partridge semen allows egg fertilization to be achieved. However, further research is required to improve sperm survival during the cryopreservation process and to optimize the AI program. From a practical standpoint, the successful cryopreservation of grey partridge semen, even as a proof-of-concept, represents a necessary first step toward the genetic management of ex situ populations. Once optimized, this technique could enable the creation of germplasm resource banks and facilitate the exchange of genetic material between distant breeding centers, minimizing stress, transport costs, and biosecurity risks associated with moving live birds. Ultimately, the future integration of frozen semen into existing conservation programs can aid in safeguarding the genetic diversity of the grey partridge.

## Ethics declaration

This study was conducted in accordance with the following guidelines for animal welfare and/or reporting: Directive 2010/63/EU on the protection of animals used for scientific purposes. Ethics committee approval was not required. The study was conducted according to Resolution No. 021/2023/P1 of 21 June 2023 issued by the Local Ethical Committee for Animal Experiments in Wrocław. Based on the activities described in the application, the Committee discontinued the proceedings, as the research involved routine veterinary procedures and standard agricultural practices related to animal husbandry. The birds were housed in facilities and enclosures designed to meet their behavioral needs.

## Author contributions

Marcel Bawej provided substantive input into the conception and methodology of the work, acquired, curated and analyzed the data, performed data visualization, secured funding and drafted the original manuscript. Marta Michalak contributed to the acquisition, curation and validation of the data, performed data visualization and critically reviewed the manuscript for important intellectual content. Regina Grugel contributed to data curation, formal analysis and data visualization and critically reviewed the manuscript for important intellectual content. Artur Kowalczyk contributed to the conception of the work, supervised the research, performed validation and critically reviewed and edited the manuscript. Furthermore, all authors explicitly approved the final version of the manuscript before submission and agree to be personally and publicly accountable for all aspects of the work, ensuring that any questions regarding the accuracy or integrity of the research are thoroughly investigated and resolved.

## Declaration of artificial intelligence and ai-assisted technologies in the writing process

During the preparation of this work, the authors used Gemini (Google) for language editing, translation, and improving sentence structure, and Scopus AI (Elsevier) to support literature search and overview. After using these tools, the authors reviewed and edited the content as needed and take full responsibility for the content of the publication.

## Disclosures

The authors declare that they have no known competing financial interests or personal relationships that could have appeared to influence the work reported in this paper.

## References

[bib0001] Bawej M., Michalak M., Rosenberger J., Kowalczyk A. (2026). Artificial insemination with liquid-stored and fresh semen in grey partridge (*Perdix perdix* L.). Poult. Sci..

[bib0002] Bezerra L.G.P., de Oliveira R.E.M., Comizzoli P., Silva A.R. (2024). Collection, handling, and preservation of wild bird semen: current status, challenges, and perspectives. Biopreserv. Biobank.

[bib0003] Blesbois E., Grasseau I., Seigneurin F., Mignon-Grasteau S., Jalme M.S., Mialon-Richard M.M. (2008). Predictors of success of semen cryopreservation in chickens. Theriogenology.

[bib0004] Kowalczyk A., Łukaszewicz E. (2015). Simple and effective methods of freezing capercaillie (Tetrao urogallus L.) semen. Plos One.

[bib0005] Laux A., Gottschalk E. (2025). Local sub-population dynamics of a central European grey partridge meta-population support large-scale conservation approach to halt its ongoing decline. Wildl. Biol..

[bib0006] O’Brien E., Sabater A.S., Toledano-Díaz A., Castaño C., de Pedro Aguilar G., Cubero D., Łukaszewicz E., Santiago-Moreno J. (2024). Effective use of artificial insemination as an ex situ conservation method for the capercaillie. Reprod. Domest. Anim..

[bib0007] Prieto R., Sánchez-García C., Tizado E.J., Alonso M.E., Gaudioso V.R. (2018). Mate choice in red-legged partridges (*Alectoris rufa* L.) kept in commercial laying cages; does it affect laying output?. Appl. Anim. Behav. Sci..

[bib0008] Santiago-Moreno J., Blesbois E. (2022). Animal board invited review: germplasm technologies for use with poultry. Animal.

[bib0009] Tajchman K., Ramankevich A., Drabik K., Kasperek K., Wójcik M. (2024). Welfare of gray partridge kept in adaptive aviaries. Med. Wet..

[bib0010] Witzenberger K.A., Hochkirch A. (2011). Ex situ conservation genetics: a review of molecular studies on the genetic consequences of captive breeding programmes for endangered animal species. Biodivers. Conserv..

